# Precise genome-editing in human diseases: mechanisms, strategies and applications

**DOI:** 10.1038/s41392-024-01750-2

**Published:** 2024-02-26

**Authors:** Yanjiang Zheng, Yifei Li, Kaiyu Zhou, Tiange Li, Nathan J. VanDusen, Yimin Hua

**Affiliations:** 1 Key Laboratory of Birth Defects and Related Diseases of Women and Children of MOE, Department of Pediatrics, West China Second University Hospital, Sichuan University, Chengdu, Sichuan 610041 China; 2 Department of Cardiovascular Surgery, West China Hospital, Sichuan University, Chengdu, Sichuan 610041 China; 3 Department of Pediatrics, Herman B Wells Center for Pediatric Research, Indiana University School of Medicine, Indianapolis, IN 46202 USA

**Keywords:** Gene therapy, Genome

## Abstract

Precise genome-editing platforms are versatile tools for generating specific, site-directed DNA insertions, deletions, and substitutions. The continuous enhancement of these tools has led to a revolution in the life sciences, which promises to deliver novel therapies for genetic disease. Precise genome-editing can be traced back to the 1950s with the discovery of DNA’s double-helix and, after 70 years of development, has evolved from crude in vitro applications to a wide range of sophisticated capabilities, including in vivo applications. Nonetheless, precise genome-editing faces constraints such as modest efficiency, delivery challenges, and off-target effects. In this review, we explore precise genome-editing, with a focus on introduction of the landmark events in its history, various platforms, delivery systems, and applications. First, we discuss the landmark events in the history of precise genome-editing. Second, we describe the current state of precise genome-editing strategies and explain how these techniques offer unprecedented precision and versatility for modifying the human genome. Third, we introduce the current delivery systems used to deploy precise genome-editing components through DNA, RNA, and RNPs. Finally, we summarize the current applications of precise genome-editing in labeling endogenous genes, screening genetic variants, molecular recording, generating disease models, and gene therapy, including ex vivo therapy and in vivo therapy, and discuss potential future advances.

## Introduction

Among the approximately 25,000 annotated genes in the human genome, over 3000 mutations have been identified in connection with diseases, and ongoing research is revealing additional genetic variations relevant to various disorders.^1^ Thus, a primary goal of biomedical research is to identify, characterize, and correct these mutations, in order to cure disease. The rapid progress in the diagnosis of genetic diseases has been driven by the decreasing costs of genome sequencing, advancements in computational techniques for comparing human genome sequences,^2^ and the expanded utilization of high-throughput genomic screening.^3–5^ Nevertheless, the scarcity of treatments, let alone cures, for genetic diseases, has led to an increasing gap between diagnostic capabilities and therapeutic options.^6^ This emphasizes a pressing need for the development of effective treatments. The prospect of mitigating or rectifying disease-causing mutations is an enticing objective with the potential to save and enhance countless lives, while the recent emergence of precise genome-editing technology provides a pathway to making the dream a reality.

**Fig. 1:**
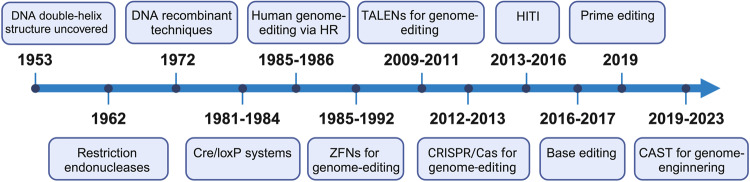
Timeline of the development of precise genome-editing tools. Key milestones in precise genome-editing are indicated. HR Homologous recombination, ZFNs Zinc-finger nucleases, TALENs Transcription activator-like effector nucleases, HITI Homology-independent targeted integration, CAST CRISPR-associated transposase. This figure was produced using BioRender.com

An ideal gene-editing technology should be able to transform a target DNA sequence into any other desired sequence while achieving high on-target editing rates (efficiency), and minimal off-target edits (specificity).^7^ The life sciences have long aspired to create gene-editing tools that possess exceptional efficiency, adaptability, product purity, and precision in targeting specific genetic sequences. In the nearly 70 years since the discovery of DNA’s double-helix structure,^8–10^ scientists have employed a variety of methods for genome modification, including homologous recombination,^11–13^ the Cre/*LoxP* system,^14–16^ zinc-finger nucleases (ZFNs),^17–19^ transcription activator-like effector nucleases (TALENs),^20–22^ and the CRISPR/Cas system and CRISPR/Cas-derived base editors (BEs) and prime editors (PEs)^23–27^ (Fig. 1). This continuous improvement and diversification of genome-editing technologies suggests that widespread correction of genetic disease via precise genome-editing is only a matter of time, and that progress towards this goal is rapidly accelerating.

This review seeks to offer a comprehensive overview of precise genome-editing modalities and their underlying mechanisms. We briefly introduce the history of precise gene editing in human disease, ranging from the earliest experiments to the development of modern techniques. Next, we discuss a variety of modern precise genome-editing strategies, highlighting the limitations and challenges of each. Lastly, we focus on therapeutic applications of precise gene editing in human disease.

## History events of precise genome-editing in human disease

The concept of utilizing gene editing for the purpose of disease treatment or trait modification can be traced back to as early as the 1950s, coinciding with the momentous uncover of DNA’s double-helix structure by Watson and Crick.^28^ In the subsequent 1960s, scientists discovered restriction endonucleases, which specifically recognize short stretches of nucleotides in DNA and create double-strand DNA breaks (DSBs) at or near the recognition locus (also known as a restriction site).^29–31^ This discovery of restriction enzymes would prove to be pivotal to the development of recombinant DNA techniques when, in 1972, Paul Berg used restriction enzymes to create the first recombinant DNA molecule, marking the birth of genetic engineering.^8,32^ In the 1980s, bacteriophage P1 Cre recombinase was discovered,^33^ and scientists soon developed mechanisms to control recombinase activity, namely through the strategic insertion of 34-nucleotide DNA sequences called “*loxP*” sites (locus of crossing, P1), which Cre will recognize and facilitate site-specific recombination between, resulting in the removal of intervening DNA.^34–37^ By the 1990s, researchers had started using the Cre/*loxP* system to engineer genomic modifications in mice.^38,39^ These ventures in genetic engineering were facilitated by the discovery that DNA sequences could be inserted into the specific loci of mammalian cells via homologous recombination (HR).^40–42^ This gene targeting technology was subsequently used on mouse embryonic stem (ES) cells which in turn were used to generate large numbers of genetically modified mouse strains.^43–48^ However, these efforts were hindered by the very low frequency of natural HR between exogenous donor DNA and target DNA.^49^

In the 1980s, scientists identified the potential to engineer zinc-finger proteins, allowing them to selectively bind to specific DNA sequences and opening the door to the design of customized DNA-binding proteins.^50–53^ The next stage in the evolution of this technology came in the late 1990s when researchers developed ZFNs capable of introducing DSBs in a sequence-specific manner. This was achieved by fusing engineered zinc-finger domains to the FokI endonuclease domain.^54^ Subsequently, ZFNs were used to produce targeted genome edits in various model organisms, including human cell lines.^55–59^ The utilization of ZFNs to induce a DSB at the target site substantially increased the efficiency of HR, with cellular success rates reported to be as high as 20%,^60^ thus greatly promoting the widespread application of gene editing. This approach of homology-directed repair (HDR) of DSBs would later become standard practice for several different genome-editing modalities. Despite the successes of ZFNs, their design and production are time-consuming, laborious, and expensive, and these factors prevented widespread adoption. In 2009, researchers discovered transcription activator-like effectors (TALEs), which, similar to zinc-finger proteins, can specifically bind to DNA sequences.^61,62^ In 2011, scientists engineered TALEs that fused to the nonspecific FokI cleavage domain (TALENs), allowing for the introduction of targeted DSBs in human cells with high efficiency.^63^ TALENs show multiple advantages over engineered ZFNs, including an easier design process, and their potential ability to be targeted to a wider range of sequences.^64^ However, this approach still suffers from the complexities associated with needing to engineer a new protein for each target.

This barrier to progress in the gene editing field would not be surmounted until 2012 when Doudna, Charpentier, et al.^23^ developed CRISPR/Cas9 gene editing systems. Unlike ZFNs and TALENs, which both specifically bind to DNA through complex engineered proteins, the CRISPR/Cas9 system relies on the specific binding of an engineered single guide RNA (sgRNA) with homology to the target DNA. These easily programmable sgRNAs bind to Cas9 and guide the Cas9 nuclease to the DNA target site, where a DSB is created. Following this discovery, Feng et al.^24^ and Church et al.^65^ employed the CRISPR/Cas9 system to achieve accurate cleavage at endogenous genomic loci in human and mouse cells. The advent of the CRISPR/Cas9 system significantly streamlined genome-editing, resulting in rapid adoption. Common simple use cases include inactivating gene function by introducing small, partially random insertions and deletions (indels), which are formed during the repair of DSBs. As with ZFNs and TALENs, precise editing can be achieved via HDR when a donor template is provided along with the guide RNA and Cas9 nuclease. However, HDR is typically restricted to dividing cells due to overlaps in the cellular machinery required for cell cycle progression and HDR.^62,66–68^ To bypass this limitation, in 2016, Belmonte et al.^69^ developed Cas9-mediated homology-independent targeted integration (HITI), enabling efficient DNA knock-in in both dividing and non-dividing cells in vitro, and notably, in vivo. While HITI can achieve robust insertion efficiencies, indel frequencies are relatively high at the junctions between the insertion and native locus, as well as at targets where insertion fails.^70^ In comparison, HDR typically results in low indel rates at positions flanking the insertion, while indels are commonly found at target sites where HDR fails.^71,72^ In addition to these limitations, all of the precise editing techniques discussed above involve the creation of DNA breaks which can trigger strong DNA damage responses, which may impact cell phenotypes.^73,74^

In 2016 and 2017, Liu et al.^25,26^ developed BEs, which are capable of chemically converting one DNA nucleotide to another at a target locus. This was achieved by fusing a nuclease-dead mutant Cas9 (dCas9) protein to a cytidine or an adenosine deaminase enzyme. The fusion protein retains the ability to be programmed with a sgRNA, but does not induce DSBs; instead, the editing complex mediates the direct conversion of C•G to T•A or A•T to G•C. This system demonstrated gene editing efficiencies up to 55%, with minimal off-target edits.^26^ However, this exciting technology is not without limitations. While BEs have the capacity to induce transition mutations, converting purine to purine (A to G) or pyrimidine to pyrimidine (C to T), they currently cannot perform the eight transversion mutations (purine to pyrimidine) and cannot perform targeted deletions or insertions. In 2019, Liu et al.^27^ further modified CRISPR/Cas9 and developed the PE system, allowing for precise modifications to DNA sequences at a specific locus through the fusion of dCas9 or nickase Cas9 with an engineered reverse transcriptase, guided by a PE guide RNA (pegRNA) specifying the target site and desired edit. This system can perform targeted insertions, deletions, and any type of point mutation, without requiring DSBs or donor DNA templates.^27^ During the same time period, Zhang et al.^75^ and Sternberg et al.^76^ characterized a CRISPR-associated transposase (CAST) that utilizes Tn7-like transposase subunits and type V-K or type I-F CRISPR effectors, enabling RNA-guided DNA transposition with unidirectional insertion of DNA segments at specific loci. Work by Kleinstiver et al.^77^ and Sternberg et al.^78^ would later demonstrate that CASTs could be engineered to precisely integrate large DNA sequences in human cells with improved integration product purity and genome-wide specificity. After nearly seventy years of development, these diverse systems showcase the rapidly evolving nature of precise genome-editing, as well as the ever-expanding number of use cases, and substantial improvements in editing efficiency and specificity (Fig. 1).

## Overview of precise genome-editing strategies

Currently, precise genome-editing is achieved using various molecular tools and techniques that activate DNA repair pathways. These techniques can be roughly divided into DSB and non-DSB mediated repair mechanisms. DSBs primarily contribute to precise genome-editing via the HDR DNA repair pathway, while a variety of genome-editing modalities utilize non-DSB DNA repair pathways (Table 1).

**Table 1:** Comparison of precise genome-editing strategies

Approach	Characteristic	Advantages	Limitations	References
HR	Point mutation, insertion, deletion; Dividing cells	High specificity	Extremely low efficiency	^40,41,49^
ZFN-HDR	Point mutation, insertion, deletion; Dividing cells	High specificity	DSB dependent; Labor intensive cloning; Low efficiency	^82,83,86,87^
TALEN-HDR	Point mutation, insertion, deletion; Dividing cells	High specificity	DSB dependent; Labor intensive cloning; Low efficiency	^64,93–96^
Cas9-HDR	Point mutation, insertion, deletion; Dividing cells	Easy to engineer	DSB dependent; PAM site necessary; Off-target effects; Low efficiency	^65,99,116,118,161^
Cre-loxP	Excision, Inversion, translocation; Dividing and non-dividing cells	High specificity; High efficiency	Not useful for insertion or correction; Need prior insertion of loxP sites	^189,194,195,197^
HITI	Insertion; Dividing and non-dividing cells	Easy to engineer	DSB dependent; PAM site necessary; Off-target effects; Low efficiency	^69,161,200^
BE	Point mutation; Dividing and non-dividing cells	High efficiency; non-dividing cells	PAM site necessary; Off-target effects; Only conversion of C•G to T•A, A•T to G•C, or C•G-to-G•C	^25,26,161,163,206,223^
PE	Point mutation, small insertion, and deletion; Dividing and non-dividing cells	Non-dividing cells	PAM site necessary; off-target effects; low efficiency; limited to small edits.	^27,247^
CAST	Large DNA insertion	Large DNA insertions	Low efficiency	^77,78^

*HR* homologous recombination, *HDR* homology-directed repair, *ZFN* zinc-finger nuclease, *TALEN* transcription activator-like effector nuclease, *HITI* homology-independent target integration, *BE* base editor, *PE* prime editor, *CAST CRISPR*-associated transposase

### HDR-mediated precise genome-editing

**Fig. 2:**
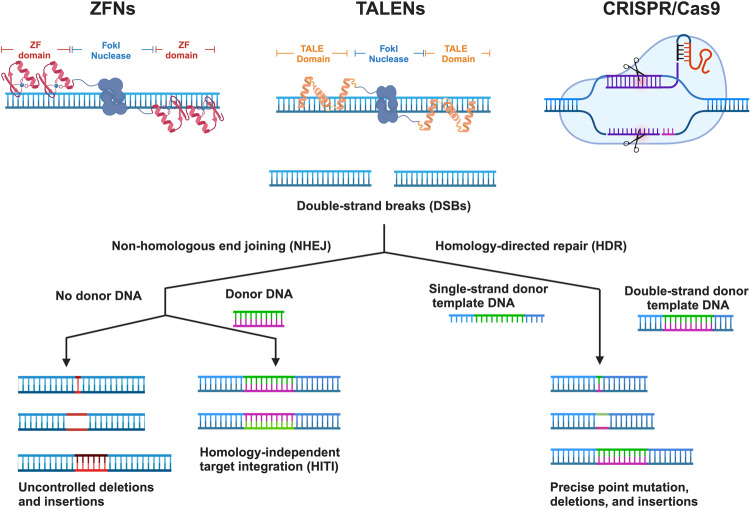
Precise genome-editing with site specific nucleases. ZFNs create double-strand breaks (DSBs) using the FokI restriction enzyme paired with specific zinc-finger DNA-binding domains. TALENs induce DSBs using the FokI restriction enzyme in conjunction with specific TALE DNA-binding domains. Cas9 nuclease, targeted by guide RNAs, creates DSBs using two distinct domains of nuclease. Genome-editing utilizing nucleases relies on two primary DNA repair pathways. The first pathway consists of end-joining mechanisms, which can be divided into classical nonhomologous end-joining (c-NHEJ), which can be used to produce targeted semi-random indels, and homology-independent target integration (HITI), which can be used to insert an exogenous sequence at a desired genomic target in the absence of homology arms. The second major repair mechanism is homology-directed repair (HDR), which primarily occurs in dividing cells, and can be used to create precise targeted edits via a single-strand or double-strand DNA donor template. ZFNs Zinc-finger nucleases, TALENs Transcription activator-like effector nucleases. This figure was produced using BioRender.com

Early methods of HR-based precise genome-editing involved the introduction of exogenous double-stranded DNA template into cells. Recombination between the target locus and the template would occasionally occur, resulting in precise editing, which was successfully used to generate knock-in cell lines and gene-modified mice. However, the frequency of HR between exogenous donor DNA and target DNA in the absence of a DSB is very low,^49^ and this shortcoming precluded the use of the approach in therapeutics. However, in the presence of both an exogenous donor template and a DSB in the target locus, HR efficiency can be improved by many orders of magnitude.^57,79^ This HDR-mediated approach to genome-editing is dependent on efficient introduction of nuclease for target-specific dsDNA cleavage. Currently, the most widely used site-specific nucleases include ZFNs, TALENs, and CRISPR/Cas9 (Fig. 2).

#### ZFNs-mediated HDR

ZFNs are chimeric proteins formed by linking the endonuclease domain of the bacterial FokI restriction enzyme with an array of site-specific DNA-binding domains, sourced from zinc-finger–containing transcription factors.^54^ The zinc-finger protein, possessing DNA-binding specificity, was initially identified in 1985 within transcription factor IIIa in *Xenopus* oocytes.^52^ An individual zinc-finger domain, consisting of approximately 30 amino acids, recognizes a 3 base pair (bp) DNA sequence.^80^ When arranged in tandem, these zinc-finger domains can potentially adhere to a longer DNA sequence 9 to 18 bp in length.^81^ By leveraging an 18 bp DNA sequence, specificity could be attained within an immense 68 billion-bp DNA pool. Thus, this breakthrough enabled the precise targeting of specific sequences within the human genome, marking a significant advancement.^82,83^

Since the FokI nuclease necessitates dimerization for DNA cleavage, ZFNs are structured as a pair that identifies two sequences bordering the target site—one on the forward strand and another on the reverse strand. When the ZFNs bind on both sides of the site, the FokI domains within the pair dimerize, leading to DNA cleavage at the target site. This process results in the creation of a DSB with 5’ overhangs.^18,84^ These DSBs can be used in combination with exogenous or endogenous donor templates to achieve precise genome-editing via the HDR pathway. Given the modular design of ZFNs, it’s possible to fine-tune the zinc-finger and nuclease domains independently. This capability empowers scientists to create new modular combinations, optimizing their affinity and specificity for applications in genome engineering.^85^

Nevertheless, ZFNs as first-generation gene editing tools have limitations. Assembling zinc-finger domains to bind an extended DNA sequence is a challenging task. The complexity of this process has hindered the dissemination of the approach beyond a small, specialized field.^86^ An additional drawback lies in the restricted target site selection, as the existing ZFN components can only address sites occurring at approximately 200 bp intervals in a random DNA sequence.^87^ This may present challenges for targeting particular sites, thus limiting the technique’s potential as a therapeutic tool.^87^

#### TALENs-mediated HDR

TALENs are also chimeric site-specific nucleases, in which an engineered array of TALE-specific DNA binding domains are fused with *FokI* endonuclease.^63,88^ TALEs, derived from plant pathogenic bacteria of the *Xanthomonas* genus, consist of repetitive sequences with 10 to 30 tandem arrays, enabling them to bind and identify extended DNA sequences.^61,89^ Each repeat comprises 33–35 amino acids, and specificity for one of the four DNA base pairs is determined by two adjacent amino acids, known as the repeat-variable di-residue.^90–92^ Thus, each repeat corresponds precisely to a base pair within the target DNA sequence. TALENs, akin to ZFNs, have the capability to induce DSBs at a specific target locus, facilitating precise genome-editing through the HDR pathway.

In comparison with ZFNs, TALENs have several potential advantages. First, recognizing individual bases with TALE–DNA binding repeats provides more design flexibility compared to triplet-confined zinc-finger proteins. TALENs can be rapidly designed and assembled in as little as two days and can be produced in large quantities, reaching into the hundreds at once.^64,93,94^ Second, the TALE repeat array can be easily extended to any desired length, in contrast to engineered ZFNs, which typically bind sequences of 9 to 18 bp.^87^ Additionally, TALENs offer more flexibility in selecting target sites, as theoretically, numerous TALEN pairs can be designed for each base pair within any arbitrary DNA sequence.^94^ Nonetheless, widespread TALEN adoption faces multiple challenges, one of which is the repetitive structure of TALENs, which may hinder their efficient packaging and delivery using certain viral vectors.^95^ Another commonly agreed limitation with TALE arrays is that TALE binding sites need to be initiated with a thymine base in order to achieve maximal binding.^96^

#### CRISPR/Cas-mediated HDR

The classical CRISPR/Cas system, which was first developed as a gene-editing tool in 2012,^23^ employs the Cas9 endonuclease. CRISPR/Cas9 system has two components: an engineered sgRNA derived from the mature tracrRNA:crRNA complex, which forms base pairs with target DNA, and the Cas9 endonuclease, which cuts the target dsDNA to create DSBs.^23^ The sgRNAs have two key features: a 20 bp sequence at the 5’ end that determines the target DNA site via Watson-Crick base-pairing, and the remaining 3’ sequence that recruits Cas9. After being guided to the target DNA sequence by the sgRNA, Cas9 recognizes the protospacer adjacent motif (PAM), an NGG sequence motif adjacent to the target, and subsequently, uses its RuvC and HNH domains to cleave the two single-strand DNA (ssDNA) sequences, forming a DSB.^28^ The HNH domain of Cas9 cleaves the DNA strand complementary to the sgRNA, and the RuvC domain cleaves the remaining strand, leading to a blunt-ended break, although DSBs with 5’ overhangs have also been proposed.^97,98^

Unlike ZFNs and TALENs, which demand protein recoding with substantial DNA segments (ranging from 500 to 1500 bp) for each unique target location, CRISPR-Cas9 offers great adaptability, as targeting is achieved by simply modifying the 20 bp protospacer sequence in the sgRNA. This is often accomplished via single-step cloning of the 20 bp segment into a plasmid encoding the sgRNA.^65^ Another potential benefit of CRISPR-Cas9 is its capability for multiplexing—utilizing multiple sgRNAs concurrently to target numerous sites simultaneously within the same cell, which is particularly valuable for assessing genetic interactions, and when generating an extensive array of vectors for targeting numerous sites or even entire genome-wide libraries.^99–102^ Thus CRISPR/Cas9-mediated HDR has been widely applied in various cultured cell lines,^103–106^ mice,^107–109^ pig,^110,111^ rabbit,^112,113^ and zebrafish.^114^

Nevertheless, A clear disadvantage of CRISPR/Cas9 is off-target nuclease activity, which can lead to serious adverse effects. To address this deficiency, one strategy involves utilizing a modified Cas9 variant capable of inducing a single-strand nick in the target DNA, as opposed to a DSB. By employing a pair of these “nickase” CRISPR-Cas9 complexes, each with binding sites on opposite DNA strands flanking the target site, allows for the generation of an outcome similar to a DSB with 5’ overhangs.^115–117^ Since a DSB is only formed when both distinct sgRNA/Cas9 complexes act at the same target, off-target activity is much less likely to result in a DSB. In another similar approach, fusion of dCas9 with the catalytic domain of Fok1 has been employed to enhance the precision of DSB creation.^118,119^ The catalytic domain of the Fok1 nuclease is only active when it forms a homodimer. Consequently, the synchronized recruitment of two Fok1 catalytic domain monomers to adjacent DNA sites is crucial for efficient and precise DNA cleavage in human cells, leading to minimal off-target editing efficiency for these systems.^118,119^ In addition to these nuclease-focused optimizations, engineering approaches for sgRNA, such as truncation or chemical modification, have demonstrated the ability to decrease off-target editing efficiency by up to three orders of magnitude while preserving high levels of on-target editing.^120,121^ Another challenge of utilizing the CRISPR/Cas9 system is the size of the Cas9 protein. The cDNA that encodes classical *S. pyogenes* Cas9 (spCas9) is approximately 4.2 kilobases (kb) in size, slightly larger than a TALEN or ZFN. This makes spCas9 challenging to deliver via commonly used viral vectors, such as adeno-associated virus (AAV), which has a cargo size limited to less than 4.7 kb.^122^ To address this shortness, novel Cas variants have been developed, such as *S. aureus* Cas9 (saCas9),^123^ Cas12a,^124^ Cas12e,^125^ Cas12f,^126,127^ Cas12j,^128,129^ and Cas12n.^130^ These new Cas variants usually have a small molecular weight. For instance, Cas12f is one of the most compact Cas variants, consisting of ~400–700 amino acids. Several groups have developed a series of Cas12f proteins, which showed efficient gene editing.^131–133^ This size reduction makes it possible to package the Cas nuclease, a guide RNA, and a donor template for HDR all within a single AAV vector.

#### The Mechanism of HR

DSBs can undergo repair through the endogenous repair machinery, involving either the non-homologous end joining (NHEJ) or HDR pathways. NHEJ introduces semi-random indels; however, when a donor DNA template, either double-stranded or single-stranded, is available and possesses homology to the adjacent sequences surrounding the DSBs, the HDR pathway may be taken, resulting in a DSB repair that follows the base sequence of the donor template, and thus achieving a precise edit. The most common form of HDR is HR, which includes two sub-pathways of double-strand break repair (DSBR) and synthesis-dependent strand annealing (SDSA).^134,135^

The initial process of HR involves the resection of a DSB to provide long 3’ single-stranded DNA overhangs. The Mre1, Rad50, and Nbs1 proteins form a three-subunit Mre1-Rad50-Nbs1(MRN) complex that recognizes and binds to the DSB ends through facilitated diffusion. The MRN complex recruits and activates the ataxia-telangiectasia mutated (ATM) protein kinase.^136,137^ Activated ATM can phosphorylate the C-terminal binding protein interacting protein (CtIP),^138^ which then interacts with breast cancer-associated protein 1 (BRCA1) to form a BRCA1/MRN/CtIP complex.^139^ The complex is then involved in 5’ end cleavage near the DSB site to expose long 3’ ssDNA overhangs.^140–142^ The overhangs are then recognized and bound by the replication protein A (RPA), which protects and stabilizes them.^143^ Subsequently, RAD51 interacts with BRCA2 to form a presynaptic nucleoprotein filament complex which replaces RPA on the ssDNA and searches for endogenous or exogenous homologous DNA.^144,145^

An intermediate displacement loop (D-loop) is formed when one of the long 3’ ssDNA overhangs invade the double stranded donor template. Next, DNA polymerase δ (Poly δ) uses the 3’ end of the invading strand to prime the synthesis of a new strand.^146–148^ In DSBR, after one strand invasion and new DNA synthesis, the second 3’ ssDNA overhangs will be captured to form two intermediates with Holliday junctions (HJs).^149^ These are accompanied by gap-filling DNA synthesis and ligation.^150^ Finally, the resolution of HJs is processed to generate either non-crossover or crossover products.^151^ Alternatively, in SDSA, the invaded template strand dissociates from the D-loop during new DNA synthesis.^152^ The newly synthesized ssDNA pairs with the complementary ssDNA strand at the opposite end of the DSBs, and the resulting ends are extended through gap-filling DNA synthesis and ligated, generating only non-crossover products.^151^ Since HDR utilizes donor templates for guiding repair, it can be harnessed to achieve precise DNA editing. However, the activation of the key protein ATM in the HDR pathway is cell cycle-dependent.^153^ Therefore, HDR is restricted in the S/G2 phases of the dividing cells.

Although HDR-mediated precise genome-editing has tremendous use cases and potential in biology and medicine, there are still limitations and challenges that need to be addressed. First, HDR is a less efficient DNA repair pathway than NHEJ.^79,154–156^ Even with recent advancements, current approaches for rectifying point mutations through HDR in therapeutically relevant settings still suffer from inefficiency.^28^ The efficiency of HDR repair depends on several factors, such as the NHEJ and HDR pathways, the length of the donor template, and the location of the target site. Moreover, achieving efficient delivery of the donor template to target cells or organisms poses a challenge, particularly for certain cell types. Second, the availability of the cellular machinery involved in HDR is typically limited to the S and G2 phases of the cell cycle.^157,158^ The cell cycle dependence of HDR can limit the efficiency of genome-editing, particularly in cells with a short S/G2 phase or in vivo applications, which often involve cells with low proliferation rates. Intriguingly, we and other teams have found that postmitotic cells can also repair DSBs through HDR when donor templates are delivered via AAV.^72,159,160^ More importantly, we found that HDR efficiency in postmitotic cells is considerable and can be comparable with mitotic cells.^72^ These findings broaden the potential applications of HDR, although further research is required to comprehensively elucidate the mechanisms involved in AAV-mediated HDR in non-dividing cells. A third limitation of HDR-mediated precise genome-editing is the requirement for DSBs, which are associated with undesired outcomes. Nuclease-induced DSBs can lead to various genomic alterations, including large deletions, retrotransposon insertions, chromosomal translocations, chromothripsis, and activation of p53, potentially resulting in the formation of oncogenic cells.^161–168^ Furthermore, the delivery of nuclease reagents to target cells or tissues is a crucial step for successful genome-editing. Delivery efficiency depends on the cell or tissue type, the delivery method used, and the stability of the reagents in vivo. For example, in select cell lines, the delivery of RNPs can generate higher concentrations of nuclear Cas9/gRNA complex, and higher editing efficiencies, than is achieved through the delivery of plasmids or viral vectors.^169^ Unfortunately, the delivery of components in vivo remains challenging for many tissues.

Efforts have been made to enhance the efficiency of HDR-mediated precise genome-editing, aiming to address the associated challenges and limitations. These strategies include inhibition of the NHEJ DNA repair pathway,^79,170,171^ activation of the HDR DNA repair pathway,^172–176^ modification of the DNA donor templates,^72,160,177–179^ and delivery of nuclease reagents.^180–182^ While the primary emphasis is on improving the accuracy and effectiveness of DSB-mediated editing, these challenges encourage the exploration of alternative strategies for precise genome-editing.

### Non HDR-mediated precise genome-editing

**Fig. 3:**
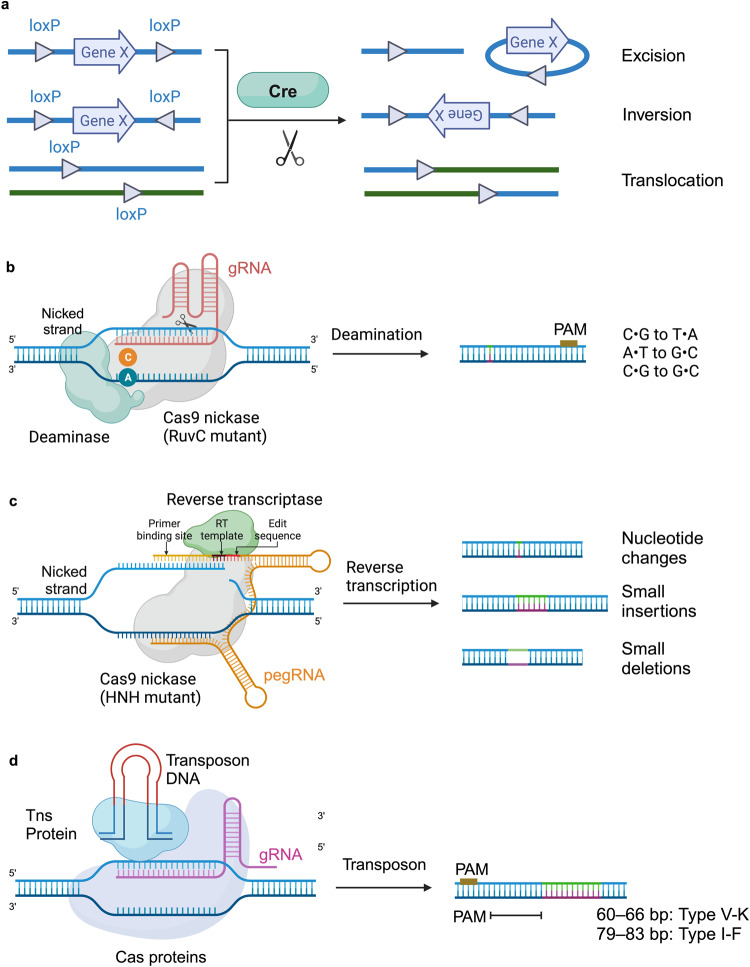
Strategies of non HDR-mediated precise genome-editing. **a** Cre/loxP system, consisting of Cre recombinase and loxP sites, facilitates DNA recombination through excision (removing a DNA segment between loxP sites in the same orientation), inversion (flipping a segment between loxP sites in opposite orientations), and translocation exchanging segments between two loxP sites in the same orientation on different DNA strands. **b** Base editing involves the introduction of C•G-to-T•A or C•G-to-G•C point mutations using cytosine base editors (CBEs), which employ Cas9 nickase or dCas9 fused to cytidine deaminase. Additionally, A•T-to-C•G point mutations can be reversed through adenine base editors (ABEs), utilizing a fusion of dCas9 or Cas9 nickase and evolved TadA* deoxyadenosine deaminase. **c** Prime editors comprise a Cas9 nickase domain fused to a reverse transcriptase domain. A prime editing guide RNA (pegRNA), engineered for specificity, directs the prime editor to its target on genomic DNA, including the desired edit within an extension. Following nicking the PAM-containing strand, the freed genomic DNA 3’ end engages in a primer–template complex with the pegRNA extension. Subsequently, the reverse transcriptase domain copies the template from the pegRNA extension into the genomic DNA directly, facilitating the addition of point mutations, small deletions, or small insertions at the target locus. **d** CAST combines Cas proteins with transposase-associated components. Transposase proteins (Tns) bind to transposon DNA, while Cas proteins are guided to the target locus in a PAM-dependent, RNA-directed manner. This localization facilitates transposon DNA integration at the target site, with each Cas-transposase complex having a specific guide RNA length and a preferred integration distance 3’ of the PAM. This figure was produced using BioRender.com

Non HDR-mediated precise genome-editing uses a diversity of DNA repair mechanisms. These strategies include site-specific recombinase systems, HITI, BEs, PEs, and CAST (Fig. 3).

#### Site-specific recombinase system

The classical site-specific recombinase system is Cre/*loxP*, which is widely employed for introducing conditional changes to transgenes and integrating DNA cassettes into eukaryotic chromosomes.^15,16,183,184^ The Cre/*loxP* system consists of two components: a Cre recombinase and specific 34 bp sequences known as *loxP* sites (Fig. 3a). Cre, a 38 kDa bacterial enzyme derived from P1 bacteriophage, has the ability to recognize and cut *loxP* sites.^185^ The *loxP* sequence consists of two recombinase-binding elements, each spanning 13 bp, arranged as near-perfect inverted repeats on opposing sides of an uneven 8 bp crossover region, which plays a critical role in establishing the orientation of *loxP* sites.^186,187^ Cre initiates the recombination event by binding to the 13 bp inverted repeat regions at the *loxP* sites. This action facilitates the formation of synaptic complexes, which consist of four Cre molecules that bridge two *loxP* sites oriented in the same direction.^188^ Subsequently, the Cre complex facilitates the exchange of DNA strands between the two sites, occurring within an asymmetrical 8 bp central spacer sequence.^189^ The asymmetrical central spacer serves as a template that unequivocally dictates the ultimate orientation of the DNA product, ensuring that the chromosomal rearrangements resulting from Cre-mediated recombination are entirely foreseeable.^189,190^ Hence, depending on how the *loxP* sites are oriented, Cre-mediated recombination can achieve excision (deletions), inversions, or translocations (Fig. 3a).^191^ In addition to the Cre/*loxP* system, various recombinase systems, such as Flp/*FRT*,^34^ Dre/*rox*,^192^ and Vika/*vox*^193^ have been developed as precise genome-editing tools.

Since site-specific recombinases mediate highly efficient and controlled DNA recombination, these systems have found widespread use in creating genetically engineered mouse models.^194–196^ However, they also have significant drawbacks. First, this recombination system cannot be used to precisely correct mutations, or to insert exogenous DNA sequences, and therefore cannot be used for gene therapy. Another drawback is that the target recognition site, such as *loxP* and *rox*, must be initially inserted into the genome, usually through techniques based on HR. Hence, the process is costly and time-consuming, and these challenges are exacerbated when aiming for more complex models involving multiple alleles.^197^

#### HITI

HITI is a NHEJ-based targeted gene knock-in method, which requires a site-specific nuclease such as Cas9 for DSB creation (Fig. 2). During NHEJ-mediated DSB repair, an exogenously supplied donor sequence gets inserted into the break site.^198,199^ In the HITI approach, the donor plasmids are designed without homology arms, preventing DSBs from being repaired via the HDR pathway. Instead, the donor DNA is designed to contain Cas9 cleavage sites flanking the donor sequence.^69^ Cas9 subsequently induces DSBs in both the genomic target sequence and the donor plasmid, resulting in blunt ends for both the target and donor sequences. The linearized donor DNA plasmid is then utilized for repair via the NHEJ pathway, facilitating its integration into the DSB site.^69^ Upon successful integration of the donor DNA into the genome in the desired orientation, it disrupts the Cas9 target sequence, thereby preventing subsequent Cas9 cleavage. In cases where the genomic DSB is repaired through error-free NHEJ without the insertion of donor DNA, the Cas9 target sequence remains intact, leading to a second round of Cas9 cleavag.^200^ As NHEJ is active throughout the cell cycle, it is noteworthy that even non-dividing cells retain their NHEJ capabilities. Therefore, HITI-mediated precise genome-editing can be used in terminally differentiated and post-mitotic cells, such as cardiomyocytes of the heart, or neurons of the brain. Indeed, HITI has been widely used for both in vitro and in vivo precise genome-editing, including gene therapy,^69,201,202^ and cell tracking.^199,203,204^ Nevertheless, HITI still has several major barriers to further adoption. A significant hurdle lies in the efficiency of current HITI methodologies. While HITI can integrate DNA at specified target sites in numerous non-dividing tissues, its efficiency often falls below 5%.^200^ Additionally, HITI can only be applied for the insertion of exogenous DNA at a target locus, but cannot be used for DNA substitution, which is necessary for correction of many mutations.^200^ Consequently, the range of genetic anomalies that HITI technology can address remains restricted. Furthermore, as discussed above, HITI also involves the creation of DSBs and off-target effects which may result in adverse consequences for genome stability.

#### BEs

BEs are capable of accurately introducing specific point mutations without requiring DSBs, DNA templates, or reliance on HDR.^25,26,205^ BEs consist of a CRISPR-Cas nuclease that has been rendered catalytically inactive, such that it only acts as a genome targeting module. These nuclease components are linked with a ssDNA deaminase enzyme and, in certain circumstances, associated with proteins that modulate DNA repair mechanisms (Fig. 3b).^25,26^ The categories of BEs are cytosine BEs (CBEs), which enable the alteration of C•G-to-T•A base pairs,^25^ adenine BEs (ABEs), which facilitate the conversion of A•T-to-G•C base pairs,^26^ and recently developed C-to-G BEs (CGBEs), which cause C•G-to-G•C base transversions (Fig. 3b).^206–208^ In BEs, the catalytically deficient Cas nuclease precisely positions an ssDNA deaminase enzyme at a specified genomic target sequence. When Cas binds, the sgRNA spacer pairs with the target DNA strand, causing the displacement of the genomic DNA strand containing the PAM and forming a ssDNA R-loop.^209,210^ CBEs employ cytidine deaminases to change cytosine bases found in the R-loop into uracils, which are then recognized by polymerases as thymine.^25,211^ ABEs utilize engineered TadA* deoxyadenosine deaminases to convert adenosine bases within the R-loop into inosines, which are recognized by polymerases as guanines.^26^ CGBEs function similarly to CBEs but promote the substitution of deaminated cytosine with guanine in the R-loop, although typically with lower efficiencies and product purities in comparison to CBEs and ABEs.^206–208^ Effective modification of target nucleotides situated within the R-loop depends on the successful interactions between the deaminase enzyme and the substrate nucleotides. This interaction within the R-loop defines the “base editing activity window”, as it is crucial for achieving efficient base editing results. In situations involving typical CBEs and ABEs employing Cas9, this activity window generally encompasses positions 4 to 8 within the protospacer (with the first nucleotide of the protospacer designated as position 1 and the PAM found at positions 21–23).^25,26^

Compared to Cas nucleases, BEs demonstrate significantly higher efficiency, generate few indel byproducts, and result in considerably fewer unintended effects associated with DSBs in direct side-by-side assessments.^161,163,168,212–214^ Thus, BEs have been widely applied in diverse cell types and organisms to introduce or reverse transition point mutations.^215–222^ Nevertheless, several limitations of BEs should be addressed. First, BEs typically deaminate nucleotides within a limited 4–5 nucleotide (nt) window, and this can lead to “bystander editing”, where adjacent C or A nucleotides near the target C or A may also undergo conversion.^25,26^ When BEs are used to modify the coding sequence, the changes typically lead to synonymous mutations within the usual base editing activity window, mainly because transition mutations often don’t affect the genetic code. Second, the effectiveness of BEs is constrained by the Cas domain’s targeting scope, which necessitates the existence of a PAM sequence at a particular distance range (usually 13 to 17 nucleotides) from the target base. Third, it’s worth noting that certain BEs may lead to off-target mutations in both DNA and RNA.^223–225^ Although engineering endeavors have alleviated numerous of these limitations.^226–231^ Additionally, current BEs are capable of inducing only 6 out of the 12 potential categories of point mutations, which means that the majority of transversions, and numerous other types of DNA edits, including insertions and deletions, remain beyond the scope of BEs.^25,26,206^

#### PEs

PEs are chimeric proteins formed by combining a Cas9 nickase domain, which is a deactivated HNH nuclease, with a laboratory-evolved Moloney murine leukemia virus reverse transcriptase (MMLV-RT) domain (Fig. 3c).^27^ The PEs are directed to the editing site using an engineered pegRNA, which includes the Cas9-binding spacer sequence, and a reverse transcriptase (RT) template that carries the intended modification and a primer binding site (PBS).^27^ Upon binding of the PE to the target site, the Cas9 nickase generates a cut in the non-target DNA strand, revealing a 3’ ssDNA segment that forms a hybrid with the PBS. This hybridized structure allows the associated RT to elongate the nicked 3’ ssDNA through the RT template (Fig. 3c). This RT activity leads to the formation of two redundant ssDNA flaps: a 5’ flap containing the original unedited sequence and a 3’ flap with the edited sequence. Although the thermodynamically favored pairing of the fully complementary 5’ flap with the unedited strand is anticipated, its vulnerability to excision by endogenous structure-specific endonucleases frequently leads to the hybridization of the edited 3’ flap, producing a heteroduplex. Eventually, the resolution of the heteroduplex involves ligation and DNA mismatch repair mechanisms, which replicate information from the edited strand to the unedited strand, ensuring the enduring integration of the desired modification. The PE system comprises three characterized versions. PE1 involves the fusion of the Cas9 nickase with the regular MMLV-RT. In PE2, the conventional MMLV-RT is replaced with an engineered pentamutant MMLV RT, resulting in a 3-fold enhancement in editing efficiency. Lastly, PE3 combines the PE2 fusion protein with pegRNA and an extra sgRNA that directs the nicking of the non-edited strand, leading to an additional 3- to 4-fold increase in editing efficiency.^27^

PEs present a distinctive array of benefits for precise genome-editing. In contrast to BEs that can only create 6 specific types of point mutations, PEs can generate all 12 varieties of single- or multi-base substitutions, small insertions, small deletions, and combinations of these modifications.^27^ Furthermore, PE has the capability to modify bases located at a considerable distance (at least 33 bp) from the initial nick created by the prime editing. Consequently, PE offers increased adaptability in contrast to base editing, as it does not require the existence of a PAM sequence near the targeted editing site. Compared to HDR, which mainly workes in the S/G2 phase of mitotic and meiotic cells, PE can be employed in non-dividing cells, which is necessary for many in vivo applications. Additionally, PE typically results in significantly fewer indel byproducts, and notably, it infrequently causes alterations in DNA at unintended off-target genomic sites. Based on these advantages, PEs have been proven to facilitate precise gene modifications in a range of cell types,^232–235^ organoids,^236,237^ zebrafish,^235^ mice,^238–242^ and plants.^243–246^ Nonetheless, the technology is still in its nascent phase, and there are several challenges that need to be addressed for the technology to fully realize its potential. First, the challenge of low editing efficiency is a critical issue, with efficiency often falling below 20% in cell lines and diminishing further in primary cells.^247^ Many endeavors have focused on effector proteins,^248,249^ pegRNAs,^250,251^ DNA repair pathways,^248,252^ and chromatin accessibility^253,254^ to improve the efficiency. Moreover, successfully delivering prime-editing reagents into the desired target cells is still an obstacle. The substantial size of the length PE hinders its integration into a single AAV vector, posing a significant challenge to its safe in vivo delivery.

#### CAST

Transposases are self-contained enzymatic systems responsible for incorporating or removing DNA segments in the genome.^255^ They function by identifying and removing the left-end (LE) and right-end (RE) motifs that flank the transposable element.^256^ The transposable element is subsequently integrated into new non-homologous sites.^255^ As a consequence of transposition, duplications of the transposon’s end sequences arise from the repair of ssDNA gaps formed during the integration of the transposon into the genome.^257^ Recent computational examination of bacterial genomes revealed the presence of CRISPR loci containing Cas genes, CRISPR RNA array elements, and transposase-specific genes.^258–260^ This finding implied the possibility of RNA-guided transposition in bacteria.^261,262^ Subsequent investigations effectively recreated simplified RNA-guided transposition utilizing type I-F and type V-K CRISPR-associated transposase (CAST) systems, leading to the targeted integration of substantial DNA fragments within bacterial genomes.^75,76,263^

The type I-F CAST system comprises three key elements: transposase operon, CRISPR-Cas-associated machinery, and the donor LE–cargo–RE transposase DNA substrate (Fig. 3d). The primary distinction between the type I-F and type V-K systems lies in their CRISPR–Cas-associated components. The type I-F system relies on the CRISPR-associated complex for antiviral defense (CASCADE) lacking the Cas3 nuclease–helicase, whereas the type V-K system CASCADE utilizes Cas12k effectors with naturally inactivated nuclease domains.^264^ In the type I-F system, transposition cargo insertions take place approximately 47–51 bp downstream from the end of the protospacer (Fig. 3d), and the optimal cargo size is determined to be around 775 bp.^76^ In comparison, insertions for type V-F occur predominantly between 60 and 66 bp downstream of the PAM sequence (Fig. 3d), enabling the integration of cargo DNA segments 500 bp to 10 kb.^75^ Comparative studies indicate that the type I-F system exhibits higher efficiency and purity of products compared to the type V-K system.^265,266^ Moreover, the type I-F system did not consistently yield detectable off-target effects throughout the *E. coli* genome, while off-target transposition of type V-K was detected at multiple loci.^267^ While the original type I-F and type V-K CAST systems were initially confined to bacterial applications, recent investigations have demonstrated the potential of engineered versions of both type I-F and V-K systems for facilitating transposon-mediated integration in human cells.^77,78^ CASTs show promise for precise genome-editing, although further engineering endeavors will be needed to improve the efficiency of integration.

## Delivery of precise genome-editing reagents

**Fig. 4:**
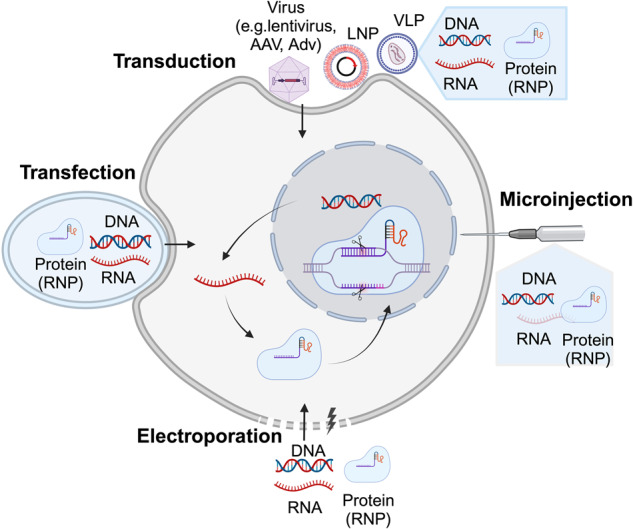
Delivery strategies for precise genome-editing reagents. Precise genome-editing components encompass a variety of forms, including DNA, RNA, and protein complexes such as ribonucleoproteins (RNPs). DNA is commonly delivered through microinjection or electroporation of plasmids, as well as viral vectors such as lentivirus, adeno-associated virus (AAV), and adenovirus (AdV). RNA can be introduced through microinjection or electroporation of RNPs, or via carriers like lipid nanoparticles (LNPs) and virus-like particles (VLPs). Proteins, specifically RNPs, are typically delivered through microinjection or electroporation, or using carriers like LNPs and VLPs. This figure was produced using BioRender.com

Efficient and safe and delivery of precise genome-editing components to target tissues is a critical prerequisite for the success of genome-editing procedures. Typically, genome-editing delivery strategies are categorized into DNAs, RNAs, and proteins, including ribonucleoproteins (RNPs) (Fig. 4).

### Delivery via DNA

The strategy for delivering DNA components via vectors such as plasmid or recombinant virus is widely used in vitro and in vivo due to simplicity (Fig. 4). In numerous cultured mammalian cell lines, high editing efficiencies can be attained by transiently transfecting cells with lipids or using electroporation to introduce genome-editing plasmids, including those for HDR, HITI, BEs, and PEs.^27,56,77,104,268–271^ While plasmid-based delivery offers convenience, one main limitation of plasmid delivery is that transfection efficiency varies by cell type and is generally limited to in vitro applications.^272,273^ An additional limitation is that delivering DNA increases the potential for non-specific DNA recombination with the genome.^274–277^ In comparison to plasmid vectors, utilizing viruses to transport DNA encoding genome-editing represents a promising delivery approach for in vivo research and therapeutic applications. The utilization of non-integrating vectors like AAV, adenoviral (AdV) vectors, or herpes simplex virus (HSV) minimizes the risk of integration of foreign DNA into the host genome. Nevertheless, infection with AdV and HSV-1 may trigger inflammatory reactions,^278^ while AAV is considered to be both non-pathogenic and non-inflammatory.^279^

Interestingly, the AAV genome is initially ssDNA and gets converted to dsDNA only after arriving in the host cell nucleus.^280^ AAV has high transduction efficiency in both mitotic and postmitotic cells, has an attractive safety profile, and the capability to specifically transduce a range of tissues via different serotypes.^122,280^ As a result, AAV is an ideal vector for genome-editing reagents delivering, which can be used for both in vivo and in vitro experiments. However, AAV is constrained by its capacity to package DNA cargo, limited to approximately 4.7 kb in length, rendering it insufficient for packaging larger genome-editing reagents, such as the Cas-mediated HDR Cas protein and donor template components (>5 kb), HITI (>5 kb), BE (~6 kb), PE (~7 kb), and CAST (>6 kb). Multiple groups have used dual AAV systems to circumvent this limitation. There are two types of dual AAV systems: in one system the first AAV carries the Cas nuclease, while the second AAV carries the HDR donor or HITI donor template respectively.^69,72,281–286^ In the second type of dual AAV delivery system large single gene editing components, such as BEs and PEs, are split between two AAV vectors, and each half is fused to a trans-splicing intein.^109,216,240,242,287–293^ Upon co-infection with these split AAVs, the large cargo protein is reassembled through a process of trans protein splicing. While this approach holds potential, the requirement for successful production and delivery of two different AAVs adds complexity and requires fine-tuning to ensure effective delivery of genome-editing reagents in living organisms.^7^

### Delivery via RNA

RNA-based delivery of genome-editing components has several promising features, including smaller molecular structures, rapid onset, and mitigation of off-target effects that are caused by persistent expression of Cas proteins.^294,295^ The delivery of genome editors in the form of mRNA is currently a widely pursued strategy. Delivering of mRNA encoding the genome-editing reagents can be achieved via various approaches, such as microinjection or electroporation, and by using non-viral vectors such as lipid nanoparticles (LNP) and newly developed virus like particles (VLPs) (Fig. 4). Microinjection or electroporation of genome editor RNAs can effectively facilitate precise genome-editing in a variety of settings, including cultured cell lines,^248,250,296^ primary human T cells,^297^ mouse embryos,^274^ mouse zygotes,^298^ and human stem cells,^299,300^ and often exhibits higher efficiency than plasmid transfection. Nonetheless, it’s worth noting that electroporation, while effective for gene editing, has been associated with negative impacts on cell viability, as the stress imposed on cells during the process can lead to alterations in gene expression profiles, potentially diminishing their proliferative capacity and modifying cellular functions.^301^ LNP technology stands as an effective method for delivering genome-editing RNAs, offering key benefits such as biodegradability, excellent biocompatibility, structural flexibility, low toxicity and immunogenicity, and high delivery efficiency and robust RNA protection against degradation.^302–307^ Multiple groups have used LNPs to deliver genome-editing RNAs, such as Cas-mediated HDR components and BEs for both in vitro and in vivo applications.^180,308–310^ Finally, VLPs, homologs of the retroviral capsid protein which are capable of binding and trafficking RNA, are an emerging approach with potential for enabling precise and effective intracellular delivery of cargo mRNAs in mammalian cells,^311–313^ but have not yet been utilized for genome-editing.

### Delivery via RNPs

RNPs-based delivery is a simple method that offers precise control over nuclease dosage without signal amplification.^314^ RNPs delivery streamlines the genome-editing process by bypassing the need for transcription and translation processes. As a result, RNPs delivery initiates genome-editing almost immediately, typically within about 3 h, and undergoes rapid degradation, usually occurring within approximately 24 h. In contrast, plasmid delivery has a longer onset time, usually taking more than 8 h to commence genome-editing, and its effects persist for several days.^315^ Moreover, RNP transfection avoids DNA integration, and minimizes the risk of off-target effects.^315^ Delivery of RNPs for precise genome-editing can be achieved using direct microinjection or electroporation, LNPs, and VLPs (Fig. 4). Microinjection or electroporation of RNPs containing precise genome-editing components, including HDR, HITI, BEs, and PEs, have demonstrated efficient editing results in diverse cell types, including cultured cell lines,^248^ human stem cells,^178,316^ primary human T cell,^182,235^ human embryos,^317^ fibroblasts,^315^ and iPSCs.^315^ Utilizing LNPs to deliver RNPs has successfully facilitated precise genome-editing via HDR and BE, both in vitro and in vivo.^169,276,318,319^ VLPs, comprising viral proteins capable of infecting cells but devoid of viral genetic material, serve as effective carriers for RNP cargoes. They harness the efficiency and tissue-targeting benefits of viral delivery while mitigating the risks, such as viral genome integration and the extended presence of the editing agent.^320^ Several groups have successfully achieved precise genome-editing by using VLPs to deliver editing components such as Cas9, donor templates, BEs, and PEs in vitro or in vivo.^321–325^ Although successful delivery of RNPs can improve gene editing, current RNPs delivery strategies are inefficiency compared to DNA and mRNA delivery.

## Applications of precise genome-editing

### Labeling endogenous genes

The spatial and temporal specificity of gene expression governs the structure and function of higher organisms. Each organ-specific cell type has a unique gene expression profile. Disruption of this expression profile will cause abnormal structure and function of organs, which manifests as disease. Similarly, the interior of eukaryotic cells is segregated by membranes into different organelles. Their structures and functions are highly specialized, and different proteins are precisely localized to ensure the normal structure and function of the organelle. Therefore, changes in cellular functions are often accompanied or caused by changes in subcellular localization of proteins.^326^ As a result, precisely mapping the subcellular localization of proteins is critical to understanding their roles in cellular processes.

Conventionally, approaches including immunostaining and overexpression of proteins fused with epitope tags or fluorescent proteins have been widely used to study protein subcellular localization. However, these methods have significant limitations. Firstly, immunostaining commonly encounters challenges due to the absence of specific antibodies against the target protein. Moreover, immunostaining often can’t distinguish between WT and mutant proteins, particularly if the mutant protein has only a small point mutation or indel. However, even small mutations can change protein subcellular localization.^327,328^ Overexpression of tagged proteins has similar limitations, one being that the cell’s protein targeting mechanisms can be overwhelmed by high levels of exogenous protein, resulting in a subcellular localization profile that dramatically differs from that of the endogenous protein.^329,330^ For example, fluorophore tags at high intracellular concentrations can cause expressed proteins to assemble into a complex, which may result in ectopic cellular localization.^331^ Thus, overexpressed fusion proteins often show diffuse localizations, while natively expressed proteins display more nuanced staining patterns.^331^

To address these problems, precise gene editing can be employed to insert epitope tags or fluorescent proteins at endogenous loci both in vivo within somatic tissues and in vitro. Early versions of this approach were based on natural HR and were used to generate knock-in mice featuring proteins fused to epitope tags or fluorescent proteins.^332^ However, generating mice by natural HR is costly and time-consuming. HDR, which employs systems such as CRISPR/Cas9, can efficiently insert epitope tags or fluorescent proteins, to label endogenous genes in vitro and in vivo.^72,160,177,333,334^ For instance, our team performed CRISPR/Cas9-mediated HDR through systemic injection of an AAV9 vector carrying donor template to Cas9-expressing mice. We successfully integrated the red fluorescent protein mScarlet into the endogenous *TTN* and *PLN* loci, creating fusion proteins which allowed for visualization of their localization.^72^ Additionally, HITI and PEs have also been employed to tag endogenous genes.^27,69,203,248,335^ For example, Belmonte et al.^69^ utilized the HITI approach to integrate sequence coding for fluorescent proteins into endogenous genes, both in cultured cells and, significantly, in living organisms. Similarly, Liu et al.^248^ employed PEs to introduce epitope tags into native genomic sites, achieving insertion rates of up to 70% in cell culture.

In addition to labeling normal proteins, precise genome-editing can also be employed to both create and determine the localization of mutant proteins, which can give important clues as to the mechanisms by which mutant proteins cause disease. However, this approach is limited by the need to create both the mutation and the epitope tag insertion at the same time with the same donor DNA, thus necessitating that the mutation be located in close proximity to the N or C-terminus. In addition to mutations close to the native termini, frameshifting mutations that result in a nearby premature stop codon can also be created and tagged in this manner. One important caveat of this approach is that some loss of function may occur from targeted alleles that undergo NHEJ instead of HDR or HITI. PEs may avoid this issue but are limited by modest insertion lengths. While NHEJ alleles will not be tagged, disruption of their function by indels may influence the localization of a tagged allele. These confounding effects can typically be mitigated by designing sgRNAs to cut in a nearby intron or untranslated region (UTR) rather than targeting the coding sequence.

### Screening genetic variants

Creating genetic perturbations and evaluating their outcomes through functional characterization or enrichment stands as a commonly employed technique for unraveling biological pathways and mechanisms. CRISPR/Cas screening-based genetic perturbations have revolutionized the field of functional genomics, offering researchers unprecedented control and precision in manipulating the genome for a deeper understanding of gene function and regulation. To date, most CRISPR screens have relied on imprecise indel formation to create loss-of-function perturbations, however, precise genome-editing can be employed to create specific variants, which can then be functionally screened.

Multiple groups have successfully achieved functional screens through precise genome-editing tools, such as HDR,^177,336–338^ BEs,^339–344^ and PEs.^345,346^ For example, Shendure et al.^337^ utilized CRISPR/Cas9-mediated HDR to precisely edit exon 18 of *BRCA1*. They employed a diverse library of donor templates to replace a 6 bp genomic region with every conceivable hexamers or the entire exon with all potential single nucleotide variants. This approach allowed them to assess substantial impacts on transcript abundance, which could be attributed to nonsense-mediated decay and the influence of exotic splicing elements. In another pioneering study, Doench et al.^339^ used BEs in pooled screens to assay variants at endogenous loci in mammalian cells. Initially, they evaluated the effectiveness of BEs in positive and negative selection screens, accurately identifying established loss-of-function mutations in *BRCA1* and *BRCA2*. Next, they screened *BH3* mimetics and *PARP* inhibitors, pinpointing specific point mutations associated with drug sensitivity or resistance. Finally, they constructed a library of sgRNAs designed to induce 52,034 ClinVar variants in 3584 genes. Through screens conducted under cellular stress conditions, they identified loss-of-function variants in multiple DNA damage repair genes. In a final example, Cohn et al.^345^ developed a high-throughput variant classification method by adapting PEs and combining it with a strategy that allows for haploidization of any locus, thereby streamlining the interpretation of genetic variants. They applied this strategy to evaluate the functionality of genetic variants with unknown significance within *NPC1*, a gene associated with the lysosomal storage disorder Niemann–Pick disease type C1.

Despite these successes, significant limitations remain. Cas nuclease-mediated HDR exhibits restricted efficiency and reduced product purity across various cell types. BEs are constrained to C•G-to-T•A and A•T-to-G•C transition edits, and the outcomes of PEs are determined through sequencing the edited locus, limiting mutagenesis to a specific gene. Nevertheless, we anticipate that continued progress in integrating precise genome-editing systems into genetic screening workflows will streamline the discovery and characterization of novel gene variants and accelerate dissection of complex genetic pathways.

### Molecular recording

Capturing and preserving a record of cellular events through modifications to genomic DNA sequences offers a means of non-invasive surveillance, which is useful for studying intricate biological systems. In molecular recording, specific genetic elements are engineered to respond to environmental signals or cellular events, leading to changes in the DNA sequence that can be deciphered through sequencing. In contrast to Cas9-mediated NHEJ, precise genome-editing has the capability to produce specific base sequences which encode digital information. This unique feature makes it feasible to effectively record the type, duration, and sequence of cellular signals over time.

Several teams have successfully recorded molecular events using BEs^347–350^ and PEs.^351^ For example, Lu et al.^350^ utilized BEs to create a platform for manipulating and assessing cellular processes, known as DOMINO (DNA-based Ordered Memory and Iteration Network Operator). This platform allows for the direct connection of stimulus-dependent base editing to a phenotypic readout. In one configuration of the system, two guide RNAs expressed from conditional promoters are required to activate a third guide RNA, which results in expression of a genomically integrated GFP gene. In this way, the platform allows for construction of programmable AND/OR logic circuits controlling the expression of genetically encodable readouts. In another example of molecular recording, Shendure et al.^351^ created the DNA typewriter system, a sequential approach for writing information into a tandem array of truncated PE target sites. These target sites are strategically designed to allow editing of only one site at a time. To transcribe activity directional manner, short PE-mediated insertions encode the cellular signal being recorded and finalize the protospacer sequence of the adjacent target site. This conversion of the adjacent site into a viable prime editing target facilitates the next recording event. The system was utilized for encoding text messages and reconstructing cellular lineages, illustrating the potential of prime editing molecular recorders in applications for biotechnology research.

While these results are promising, the field remains at a nascent stage. One key concern is that these tools may introduce unintended mutations or alterations in the genome, leading to unpredictable outcomes, such as compromised reliability and accuracy of the recorded information.^351^ Balancing the benefits of molecular recording with the need for minimizing unintended consequences and improving the sophistication and capacity of recording systems will remain active areas of development.

### Generating disease models

Mutations that alter amino acids or nucleotides are often found in the genomes of patients with genetic diseases and are widely used in the simulation of human diseases in various model systems. Researchers utilize two key tools to mimic human disease: transgenic animals involve the random insertion of a foreign gene into an animal’s genetic material, and gene edited animals, where specific genes are either disabled or modified.^352^ In contrast to transgenic disease models, where the incorporation of foreign genes and their regulatory elements can pose a risk of disrupting host genes, potentially leading to cancer or other dysfunction,^353–358^ the precise alterations created in gene-edited animals are less likely to have unintended consequences. Early animal models were generated by introducing edited embryonic stem (ES) cells into blastocysts, a process often relying on inefficient natural HR.^359,360^ By using higher efficiency methods such as HDR, BEs, and PEs, this process has been dramatically accelerated.

Multiple research teams have successfully employed HDR, EBs, and PEs strategies to efficiently produce various disease models, including edited mice,^234,238,361–364^ rats,^363^ pigs,^365^ zebrafish,^221,235^ and *Drosophila*.^366^ For example, when Gruber et al.^361^ utilized CRISPR/Cas9-mediated HDR to generate *Mpl*^*S504N*^ mutant mice, 2 of 16 founder mice harbored the mutation and displayed the anticipated myeloproliferative neoplasms (MPNs) phenotype. Similarly, ABE9 was employed by Li et al.^363^ to achieve specific A-to-G conversions in mouse and rat embryos, efficiently creating disease models, with an impressive efficiency rate of up to 62.41%. Finally, Kim et al.^234^ harnessed enhanced prime editing to produce mutant mice, achieving editing frequencies as high as 47%.

### Gene therapy

Traditional treatments for genetic disease perform poorly, providing only limited relief of clinical symptoms in many cases. Hence, effective, and safe gene therapy methods are urgently needed to achieve a radical cure for genetic diseases. Thus far two methods of gene therapy have been widely used: first, viral delivery of an exogenous wildtype gene to replace the defective endogenous gene^367–372^ and second, use of RNA interference (RNAi)^373–376^ to degrade target mRNA and suppress the expression of defective genes. These methods, however, have limitations. For example, delivery of therapeutic genes by viral vectors might introduce new mutations during the process of virus entry into the body, resulting in dysregulation of endogenous gene expression,^377^ and non-integrating viral vectors can be lost over time,^378,379^ while RNAi may suffer from modest inhibitory effects and poor specificity.^380,381^

**Fig. 5:**
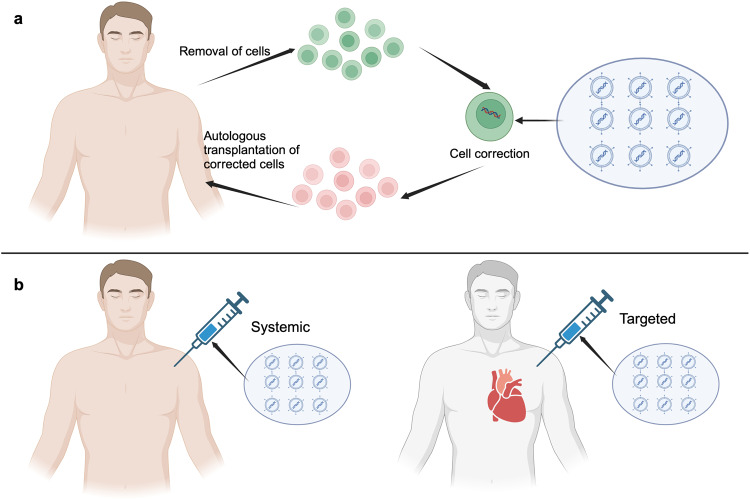
Ex vivo and in vivo precise genome-editing therapy. **a** In ex vivo editing therapy, cells are harvested from the patient, modified, and subsequently reintroduced. **b** For in vivo systemic therapy (on the left), universal delivery agents are employed, capable of targeting a broad range of tissue types. For targeted in vivo therapy (on the right), targeted intervention can be accomplished by directly injecting viral vectors into the affected tissue or through systemic administration of vectors with an innate affinity for particular tissues, such as the heart, liver, or skeletal muscle. This figure was produced using BioRender.com

Given their ability to create specific, site-directed DNA insertions, deletions, and substitutions, precise genome editors have garnered significant interest in the biomedical research community. This interest stems from the potential to correct genetic mutations associated with human illnesses. Precise genome-editing-mediated gene therapy can be performed ex vivo or in vivo (Fig. 5). In ex vivo gene therapy, the target cells are removed from the patient and cultured in vitro, where the mutant gene can be corrected via genome-editing. Subsequently, these edited cells are expanded to produce enough cells expressing the corrected gene, and finally returned to the patient (Fig. 5a). For in vivo gene therapy, the genome-editing elements necessary for genome-editing are directly introduced to the body via RNPs, LNP, or viral vectors, to make systemic or target somatic cell genome edits (Fig. 5b).^1^ Currently, various precise genome-editing strategies have been extensively employed in gene therapy for a variety of tumors and genetic disease, many of which have entered clinical trials (Tables 2 and 3).

**Table 2:** Clinical trials of precise genome-editing therapy

Disease type	Target	Strategies	Phase	Trial number
Mucopolysaccharidosis I	*Albumin*	ZFN-HDR	I/II	NCT02702115
Mucopolysaccharidosis II	*Albumin*	ZFN-HDR	I/II	NCT03041324
Relapsed Or Refractory CD19+ Leukemia and Lymphoma	*TCR*	Cas9-HDR	I	NCT05037669
Relapse/Refractory B-cell Lymphoma	*AAVS1*	Cas9-HDR	I	NCT04213469
Locally advanced or metastatic solid tumors	*TRAC*	Cas9-HDR	I	NCT03970382
Relapsed or Refractory B-Cell Malignancies	*TRAC*	Cas9-HDR	I	NCT04035434
β-thalassemia	*HBB*	Cas9-HDR	I	NCT03728322
Sickle cell disease	*HBB*	Cas9-HDR	I/II	NCT04774536
Sickle cell disease	*HBB*	Cas9-HDR	I/II	NCT04819841
β-thalassemia	*BCL11A*	BE	I	NCT06065189
β-thalassemia	*BCL11A*	BE	I	NCT06024876
Sickle cell disease, β-thalassemia	*HGB1* and *HGB2*	BE	I/II	NCT05456880
T cell malignancies	*CAR7*	BE	I	NCT05397184
Acute myeloid leukemia (AML)	*CAR33*	BE	I	NCT05942599
Acute lymphoblastic leukemia/CD7+ acute myeloid leukemia	CD7, *TRAC*, *PDCD1*, and CD52	BE	I/II	NCT05885464
Familial hypercholesterolemia	*PCSK9*	BE	I	NCT05398029

*ZFN* zinc-finger nuclease, *HDR* homology-directed repair, *BE* base editor

**Table 3:** Precise genome-editing-mediated gene therapy

Environment	Disease type	Target	Strategies	Editing efficiency	Delivery	References
Ex vivo	Sickle cell disease	*HBB*	ZFN-HDR	~18% T > A correction	Lentivirus and electroporation	^392^
			Cas9-HDR	20–30% T > A correction	AAV and electroporation	^393,394^
			ABE	40%–80% A > G correction	Electroporation	^395^
			PE	~27% T > A correction	Electroporation	^386^
	β-thalassemia	*IVS1*	ABE	~80% A > G correction	Electroporation	^396^
		*BCL11A, HBB*	CBE	~90% C > T correction of *BCL11A*, ~18.2 C > T correction of *HBB*	Electroporation	^397^
		*BCL11A, HBG*	ABE	~94.3% A > G correction of *BCL11A*, ~85.5% A > G correction of *HBG*	Electroporation	^398^
	X-linked chronic granulomatous disease	*CYBB*	ZFN-HDR	~7.1% *CYBB* cDNA insertion	AAV and electroporation	^400^
			Cas9-HDR	~21% T > C correction	Electroporation	^399^
	Lymphoblastic leukaemia	*TRAC*	Cas9-HDR	~46.5% CAR insertion of T cells	AAV6 and electroporation	^407^
	Glioblastoma	*AAVS1*	Cas9-HDR	>90% CAR insertion of cells	Nucleofection	^408^
In vivo	Duchenne muscular dystrophy	*DMD*	HITI	4% ~ 7% Exon 52 insertion	AAV9	^284^
			ABE	~51.0% A > G correction	AAV9	^287^
	Spinal muscular atrophy	*SMN2*	ABE	~37% T > C correction	AAV9	^410^
	Hypertrophic cardiomyopathy	*Myh6*	ABE	~32.3% A > G correction	AAV9	^412^
			ABE	~32.3% A > G correction of DNA	AAV9	^412^
	Ornithine transcarbamylase deficiency	*OTC*	Cas9-HDR	~10% A > G correction	AAV8	^282^
			Cas9-HDR	~6% *OTC* cDNA insertion	AAV8	^413^
	Familial Hypercholesterolemia	*LDLR*	Cas9-HDR	~6.7% T > G correction	AAV8	^283^
	Fabry disease	*GLA*	ZFNs-HDR	~1.7% human GLA cDNA insertion	AAV8	^414^
	Adrenoleukodystrophy	*ABCD1*	HITI	Human *ABCD1* insertion	AAV9	^286^
	phenylketonuria	*PAH*	CBE	21.9–26.9% C > T correction	AAV8	^109^
			PE	2.0%–6.9% C > T correction	AdV	^240^
	Type I tyrosinemia	*FAH*	Cas9-HDR	~9% A > G correction	Plasmid	^415^
			ABE	~9.5% A > G correction	Plasmid	^310^
			PE	~11.5% A > G correction	Plasmid	^417^
			PE	~0.76% 1.3 kb deletion and 19 bp insertion of hepatocytes	Plasmid	^416^
	Hutchinson-Gilford progeria syndrome	*LMNA*	ABE	20%–60% T > C correction	AAV9	^419^
	Inherited retinal disease	*RPE65*	ABE	~16% T > C correction	Lentivirus	^421^
			ABE	~22% T > C correction	AAV2	^422^
			PE	~6.8% T > C correction	AAV8	^417^
			PE	~11.4 T > C correction	AAV8	^420^
	Atherosclerotic cardiovascular disease	*PCSK9*	ABE	~50% A > G conversion	AAV8	^425^
			ABE	~60% A > G conversion	LNP	^426,427^
	Ischemia/reperfusion injury	*CaMKIIδ*	ABE	7.6% A > G conversion	AAV9	^430^

*ZFN* zinc-finger nuclease, *HDR* homology-directed repair, *HITI* homology-independent target integration, *ABE* adenine base editor, *CBE* cytosine base editor, *PE* prime editor, *AAV* adeno-associated virus, *AdV* adenovirus, *LNP* lipid nanoparticles

#### Ex vivo gene therapy

Ex vivo gene therapy has multiple advantages. First, this type of gene therapy allows the target cells to be easily manipulated via various elements (such as RNPs, mRNA, DNA, and proteins) through various delivery systems, including viral vectors, electroporation, lipid nanoparticles, cell-penetrating peptides, and carbon nanowires.^382–388^ This broad selection of delivery options often translates to high gene editing efficiencies.^382,389^ Second, ex vivo therapy is well-targeted, since only the targeted cells are present at the time of editing.^390^ Moreover, ex vivo therapy can trigger a smaller immune response in comparison to in vivo therapy since there are no gene-editing elements directly introduced into the body.^390,391^ Third, non-specific edits that result in harmful phenotypes can be screened out prior to the cells being returned to the patient, thus resulting in a much more attractive safety profile for this approach.

Precise genome editors have been successfully applied for ex vivo gene therapy to fix mutations associated with various genetic diseases of the blood system. Kohn et al.^392^, Tisdale et al.^393^, Porteus et al.^394^ and Liu et al.^386,395^ employed HDR, BEs, and PEs to correct the *HBB* gene in hematopoietic stem cells (HSCs) as a treatment for Sickle cell disease (SCD). These approaches yielded therapeutic-level gene correction with efficiency ranging from 30% to 80%. Subsequent transplantation of these modified human HSCs into immunodeficient mice resulted in a significant reduction in hypoxia-induced sickling of bone marrow reticulocytes, suggesting enduring and effective genome-editing. Miccio et al.^396^ Bauer et al.^397^ and Bauer et al.^398^ utilized ABE8e to specifically target the prevalent *HBB* mutation *IVS1-110* (G > A), *BCL11A* enhancer, or both the *BCL11A* enhancer and HBG promoters in hematopoietic stem and progenitor cells (HSPCs) from β-thalassemia patients, achieving gene editing efficiencies of up to 80%. Long-lasting therapeutic modifications were achieved in self-renewing repopulating HSCs, as evidenced by assessments in both primary and secondary recipients. Furthermore, the durable therapeutic editing extended to self-renewing repopulating human HSCs, as evidenced in primary and secondary recipient assays. In another case of successful therapeutic gene editing, Malech et al.^399,400^ achieved insertion of the wild-type *CYBB* gene into the *AAVS1* locus or corrected the mutant *CYBB* gene in CD34+ HSPCs obtained from patients with the X-linked chronic granulomatous disease (X-CGD) using HDR strategies. This HDR-mediated *CYBB* insertion or correction resulted in efficient restoration of CYBB expression and increased NADPH oxidase activity. In a final example, transplant of gene-repaired X-CGD HSPCs into C-CGD model mice led to successful engraftment and the generation of functional mature human myeloid and lymphoid cells. In addition, precise genome-editing, including HDR and HITI, can also be applied for chimeric antigen receptor (CAR)-T cell therapy. Previously, CARs were typically delivered to T cells via γ-retroviral transduction, or other randomly integrating vectors.^401–403^ However, the use of these vectors comes with potential drawbacks, including clonal expansion, variegated transgene expression, oncogenic transformation, and transcriptional silencing.^404–406^ In contrast, HDR and HITI enable efficient sequence-specific insertion of CARs, which avoids the above limitations. This approach was demonstrated by Sadelain et al.^407^ Bao et al.^408^ Hunag et al.^409^ and Feldman et al.^202^ who achieved highly efficient and precise gene targeting of CAR-T cells using HDR or HITI, resulting in enhanced T cell potency both in vitro and in vivo.

Although the advantages of ex vivo gene therapy are notable, limitations also should be considered. First, the target cells of ex vivo therapy must be able to survive outside the body for a long period of time, which is problematic for many fully differentiated cell types. As a consequence, ex vivo gene therapy is primarily constrained to tissues containing adult stem cells, such as the hematopoietic systems.^1^ A second major limitation of ex vivo therapy is that when cells cultured in vitro are transplanted back into the patient, the transplantation efficiency is often poor, which reduces the effectiveness of the treatment.^1^

#### In vivo gene therapy

Compared with ex vivo gene therapy, in vivo gene therapies have several advantages. First, in vivo therapies may be more suitable for treating cell types that cannot be readily cultured and expanded ex vivo. Second, in vivo therapies can be more cost effective since slow and labor-intensive culture processes are avoided. Third, in vivo therapies can simultaneously target a variety of tissue types, making it possible to treat diseases that affect multiple organ systems.

In vivo gene therapies based on precise genome-editing have been employed to directly correct disease-causing mutations or to precisely insert a therapeutic transgene at an endogenous locus linked to a variety of genetic disorders in humans associated with Duchenne Muscular Dystrophy (DMD),^284,287^ Spinal Muscular Atrophy (SMA),^410^ Hypertrophic Cardiomyopathy (HCM),^411,412^ Ornithine Transcarbamylase Deficiency (OTCD),^282,413^ Familial Hypercholesterolemia (FH),^283^ Fabry disease,^414^ Adrenoleukodystrophy (ALD),^286^ Phenylketonuria,^109,240^ Type I tyrosinemia,^310,415–418^ Hutchinson-Gilford Progeria Syndrome (HGPS),^419^ and inherited retinal disease.^417,420–422^ For instance, in a mouse model of OTCD, Wilson et al.^282,414^ corrected a mutant *OTC* gene or inserted a codon-optimized human OTC (hOTCco) transgene at the mutant *OTC* locus via delivery of CRISPR/Cas9-mediated HDR components with a dual AAV system. These two approaches resulted in a reversal of the mutation in 6.7% to 20.1% of hepatocytes and improved the survival of mice exposed to a high-protein diet, which typically worsens the disease. Gersbach et al.^284^ employed an AAV-based HITI method to correct the expression of full-length dystrophin in a DMD mouse model. This led to the successful correction of full-length dystrophin expression in both skeletal and cardiac muscle, effectively alleviating the disease symptoms. Schwank et al.^109^ corrected the *PAH* point mutation linked to phenylketonuria in mice using an intein-split base editing approach delivered by dual AAVs. This approach led to mRNA PAH correction rates reaching as high as 63%, restoration of PAH enzyme activity, and the reversal of the light fur phenotype in *PAH*^*enu2*^ mice. Sontheimer et al.^418^ addressed a *FAH* transversion mutation in a mouse model of tyrosinemia type I through the delivery of PEs via AAV and hydrodynamic tail-vein injection. These interventions resulted in the recovery of weight in the mice, with editing efficiencies in the liver reaching approximately 11.5% from AAV delivery and 1.3% from hydrodynamic injection.

In addition to genetic diseases, in vivo precise genome-editing also has been used in non-genetic disease, such as cardiovascular disease. PCSK9 is predominantly found in the liver and functions as an inhibitor of the LDL receptor.^423^ Inhibiting PCSK9 disrupts its binding to the LDL receptor, leading to a reduction in blood LDL levels, offering a potential treatment avenue for atherosclerotic cardiovascular disease.^424^ Liu et al.^425^ Kathiresan et al.^426^ and Schwank et al.^427^ used base editing to knockdown PCSK9 in mice and nonhuman primate models, resulting in substantial reductions of PCSK9 and LDL cholesterol levels, with approximately 90% and 60% reductions observed in mice and nonhuman primates, respectively. A second cardiovascular application relates to *CaMKIIδ*, which plays a pivotal role in regulating cardiac signaling and function.^428^ Nevertheless, prolonged CaMKIIδ overactivity is associated with various cardiac diseases in both humans and mice, such as hypertrophy, ischemia/reperfusion (IR) injury, arrhythmias, and heart failure.^429^ Olson et al.^430^ employed BEs to eliminate the oxidative activation sites of *CaMKIIδ*. Editing *CaMKIIδ* in mice during an episode of IR rescued cardiac function, even after substantial damage.

Although advances in precise genome-editing in vivo gene therapy provide examples of various therapeutic strategies, the low efficiency and the immune response caused by viral vectors should be considered. Another challenge is that the sustained expression of in vivo genome-editing reagents, such as Cas9, may increase off-target effects and genotoxicity. Thus, spatiotemporal control of CRISPR/Cas9 expression is particularly important.^431^

## Discussion and perspectives on future directions

Precise genome-editing can be achieved through a variety of distinct tools that introduce precise changes to the DNA sequence of cells or organisms. The capability to precisely manipulate the genome with precision has numerous direct applications in basic science research, disease modeling, and medicine. However, despite significant recent progress, low efficiency remains a major barrier to further adoption. BEs are less affected by this issue, demonstrating high in vivo editing efficiency, but unfortunately, BEs are limited to C•G-to-T•A and A•T-to-G•C transition edits. In addition to BEs, recent advances have significantly improved the efficiency of other precise genome-editing approaches, such as HDR and PEs, both in vitro and in vivo. While early results are promising, the efficiency of these gene editing approaches needs to be improved to enable broad development of effective gene therapies. A second major challenge of precise genome-editing is off-target mutations, particularly in systems based on CRISPR/Cas9, such as HDR, HITI, BEs, and PEs. Newly developed Cas variants with lower rates of off-target editing have been reported, such as a new subtype of Cas12f, enAsCas12f, which showed lower off-target effects than Cas9.^133^ However, the efficiency of these new Cas variants in targeting DNA needs additional improvement. Furthermore, delivery of precise genome-editing reagents to target cells or tissues is a crucial step for successful genome-editing. The delivery efficiency depends on the cell or tissue type, the delivery method used, and the stability of the reagents in vivo. For example, delivery of RNPs can be more efficient than delivery of plasmids or viral vectors.^169^ Unfortunately, delivery of components to many tissues in vivo remains challenging, and will require considerable additional vector engineering efforts.

The ongoing refinement of existing editing tools will focus on improving efficiency and specificity, while expanding targeting capabilities and optimizing delivery systems will also drive the field forward. Ultimately, these improvements will enable more sophisticated applications, including the development of novel therapies.
